# Supplementary material for: Characteristics and six-month viral load suppression of clients presenting with advanced HIV disease in South Africa
Source: PLOS Glob Public Health. 2025 Sep 23;5(9):e0004927. doi: 10.1371/journal.pgph.0004927 (PMC12456826; doi:10.1371/journal.pgph.0004927)
Supplement: S1 Table — (DOCX) [file pgph.0004927.s001.docx]

**Supplementary Table 1. Socio-demographic characteristics of PREFER study participants with or without baseline CD4 count**

| **Characteristic** | **Level** | **Patients without baseline CD4 count** | **Clients with baseline CD4 count** | **Total** |
| --- | --- | --- | --- | --- |
| N (%) |  | n= 160 (15) | n = 938 (85) | (N = 1098) |
| Median age (IQR) |  | 35 (29-42) | 33 (27-40) | 33 (27-41) |
| Age | 18-24years | 19 (12) | 169 (18) | 188 (17) |
|  | 25-49 years | 124 (78) | 677 (72) | 801 (73) |
|  | 50+ years | 17 (11) | 92 (10) | 109 (10) |
| Sex | Male | 51 (32) | 261 (28) | 312 (28) |
|  | Female | 109 (68) | 677 (72) | 786 (72) |
| Marital status | Married living with partner | 58 (36) | 289 (31) | 347 (32) |
|  | Married but not living with partner | 64 (40) | 441 (47) | 505 (46) |
|  | Single | 38 (24) | 208 (22) | 246 (22) |
| Education | Primary or less | 62 (39) | 347 (37) | 409 (37) |
|  | Secondary | 72 (45) | 453 (48) | 525 (48) |
|  | Post-secondary | 26 (16) | 138 (15) | 164 (15) |
| Occupation | Formal | 36 (22) | 204 (22) | 240 (22) |
|  | Informal | 29 (18) | 187 (20) | 216 (20) |
|  | Unemployed | 85 (53) | 477 (51) | 562 (51) |
|  | Student/Trainee | 10 (6) | 70 (7) | 80 (7) |
| Food scarcity | Never | 114 (71) | 658 (70) | 772 (70) |
|  | Seldom | 13 (8) | 59 (6) | 72 (7) |
|  | Sometimes | 29 (18) | 191 (20) | 220 (20) |
|  | Often | 4 (2) | 30 (3) | 34 (3) |
| Access to money for health care | Very difficult | 24 (15) | 127 (14) | 151 (14) |
|  | Difficult | 72 (45) | 392 (42) | 464 (42) |
|  | Easy | 57 (36) | 378 (40) | 435 (40) |
|  | Very easy | 7 (4) | 41 (4) | 48 (4) |
| Facility patient volume | <2000 TROA | 55 (34) | 236 (25) | 291 (27) |
|  | 2000-4000 TROA | 56 (35) | 371 (40) | 427 (39) |
|  | >4000 TROA | 49 (31) | 331 (35) | 380 (35) |
| Patient ART history | Initiating today | 38 (24) | 273 (29) | 311 (28) |
|  | Re-engagement | 33 (21) | 135 (14) | 168 (15) |
|  | On treatment | 89 (56) | 530 (57) | 619 (56) |
