# Supplementary material for: Characteristics and six-month viral load suppression of clients presenting with advanced HIV disease in South Africa
Source: PLOS Glob Public Health. 2025 Sep 23;5(9):e0004927. doi: 10.1371/journal.pgph.0004927 (PMC12456826; doi:10.1371/journal.pgph.0004927)
Supplement: S2 Table — (DOCX) [file pgph.0004927.s002.docx]

Supplementary table 2. Preferences and expectations AHD clients stratified by ADH disease as defined by CD4 <200 cells/ µL or WHO stage 3 or 4 conditions and CD4 <200 cells/ µL only

| **Preference** |  | Symptomatic AHD clients | Asymptomatic AHD clients |
| --- | --- | --- | --- |
| N (%) |  | 41 (15) | 234 (85) |
| Offered choice? | Yes | 3 (7) | 13 (6) |
| Frequency of clinic visits | Every month | 9 (22) | 34 (15) |
|  | Every 2 months | 10 (24) | 67 (29) |
|  | Every 3 months | 15 (37) | 96 (41) |
|  | Every 6 months | 6 (15) | 36 (15) |
|  | Other (specify) | 1 (2) | 1 (0) |
| Dispensing intervals | 1 month at a time | 8 (20) | 28 (12) |
|  | 2 months at a time | 10 (24) | 68 (29) |
|  | 3 months at a time | 16 (39) | 100 (43) |
|  | 4 months at a time | 1 (2) | 2 (1) |
|  | 6 months at a time | 6 (15) | 36 (15) |
| Part of the month | Early in the month (first week) | 17 (41) | 52 (22) |
|  | Late in the month (last week) | 7 (17) | 37 (16) |
|  | Middle of the month | 5 (12) | 45 (19) |
|  | Doesn’t matter, can come any time during the month | 12 (29) | 100 (43) |
| Day of the week | Monday | 12 (29) | 79 (34) |
|  | Tuesday | 15 (37) | 75 (32) |
|  | Wednesday | 16 (39) | 99 (42) |
|  | Thursday | 14 (34) | 81 (35) |
|  | Friday | 9 (22) | 95 (41) |
|  | Saturday | 8 (20) | 52 (22) |
|  | Sunday | 9 (22) | 34 (15) |
| Time of the day | Before work in the morning (before 8 am) | 16 (39) | 88 (38) |
|  | Mornings (8 am to 12 pm) | 19 (46) | 124 (53) |
|  | Lunch time (12 am to 2 pm) | 3 (7) | 17 (7) |
|  | Afternoons (2 to 4 pm) | 2 (5) | 16 (7) |
|  | After work in the early evening (4-7 pm) | 0 (0) | 9 (4) |
|  | Other | 1 (2) | 3 (1) |
| Accompanied to the clinic | Alone | 35 (85) | 206 (88) |
|  | With a family member | 3 (7) | 17 (7) |
|  | With a friend | 1 (2) | 5 (2) |
|  | With someone else (specify) | 0 (0) | 1 (0) |
|  | Doesn’t matter/no preference | 2 (5) | 5 (2) |
| External pick up | Yes | 21 (51) | 156 (67) |
| Home delivery | Yes | 25 (61) | 123 (53) |
| Medication packaging | One bottle for each month | 18 (44) | 99 (42) |
|  | One larger bottle with several months in it | 9 (22) | 42 (18) |
|  | An unmarked (blank) container | 2 (5) | 25 (11) |
|  | A container with instructions on it | 2 (5) | 19 (8) |
|  | A blister pack | 5 (12) | 19 (8) |
|  | Any kind of packaging is fine | 5 (12) | 30 (13) |
| Provider choice | Doctor or clinical officer | 5 (12) | 42 (18) |
|  | Nurse | 36 (88) | 178 (76) |
|  | Counsellor | 0 (0) | 10 (4) |
|  | Community health worker | 0 (0) | 3 (1) |
|  | Peer/expert patient | 0 (0) | 1 (0) |
| More information | More | 15 (37) | 123 (53) |
|  | The same | 25 (61) | 100 (43) |
|  | Less | 1 (2) | 11 (5) |
| More counselling | More | 17 (41) | 123 (53) |
|  | The same | 24 (59) | 102 (44) |
|  | Less | 0 (0) | 9 (4) |
| Information format | Written material (brochure or information) | 12 (29) | 101 (43) |
|  | Class/group session in community (not at clinic) | 1 (2) | 18 (8) |
|  | Class/group session with provider at clinic | 8 (20) | 39 (17) |
|  | One-on-one session with provider at clinic | 25 (61) | 120 (51) |
|  | Social media (e.g. Facebook, Twitter) | 2 (5) | 45 (19) |
|  | Community group in my community | 1 (2) | 7 (3) |
|  | Radio or TV | 11 (27) | 56 (24) |
|  | Videos I can watch online at home | 3 (7) | 20 (9) |
|  | Text messages on my phone | 18 (44) | 121 (52) |
|  | Links to websites that I can browse in my own time | 3 (7) | 46 (20) |
|  | Other specify | 1 (2) | 1 (0) |
| Facility care | As good as | 24 (59) | 176 (75) |
|  | Better than | 13 (32) | 51 (22) |
|  | Worse than | 4 (10) | 7 (3) |
