# Supplementary material for: Characteristics and six-month viral load suppression of clients presenting with advanced HIV disease in South Africa
Source: PLOS Glob Public Health. 2025 Sep 23;5(9):e0004927. doi: 10.1371/journal.pgph.0004927 (PMC12456826; doi:10.1371/journal.pgph.0004927)
Supplement: S3 Table — (DOCX) [file pgph.0004927.s003.docx]

Supplementary table 3. AHD clients service delivery preferences stratified by sex

| Preference | Level |  |  |
| --- | --- | --- | --- |
|  |  | Male | Female |
| N (%) |  | 121 (44) | 154 (56) |
| Offered choices? | Yes | *6 (5)* | *10 (6)* |
| Frequency of clinic visits | Every month | 24 (20) | 20 (13) |
|  | Every 2 months | 29 (24) | 48 (31) |
|  | Every 3 months | 50 (41) | 60 (39) |
|  | Every 6 months | 18 (15) | 24 (16) |
|  | Other (specify) | 0 (0) | 2 (1) |
| Dispensing intervals | 1 month at a time | 21 (17) | 16 (10) |
|  | 2 months at a time | 30 (25) | 48 (31) |
|  | 3 months at a time | 51 (42) | 64 (42) |
|  | 4 months at a time | 1 (1) | 2 (1) |
|  | 6 months at a time | 18 (15) | 24 (16) |
| Part of the month | Early in the month (first week) | 29 (24) | 40 (26) |
|  | Late in the month (last week) | 24 (20) | 20 (13) |
|  | Middle of the month | 19 (16) | 32 (21) |
|  | Doesn’t matter, can come any time during the month | 49 (40) | 62 (40) |
| Day of the week | Monday | 35 (29) | 56 (36) |
|  | Tuesday | 42 (35) | 48 (31) |
|  | Wednesday | 47 (39) | 69 (45) |
|  | Thursday | 45 (37) | 49 (32) |
|  | Friday | 46 (38) | 58 (38) |
|  | Saturday | 34 (28) | 26 (17) |
|  | Sunday | 26 (21) | 17 (11) |
| Time of the day | Before work in the morning (before 8 am) | 36 (30) | 67 (44) |
|  | Mornings (8 am to 12 pm) | 68 (56) | 76 (49) |
|  | Lunch time (12 am to 2 pm) | 8 (7) | 12 (8) |
|  | Afternoons (2 to 4 pm) | 9 (7) | 9 (6) |
|  | After work in the early evening (4-7 pm) | 4 (3) | 5 (3) |
|  | Other | 12 (10) | 4 (3) |
| Accompanied to the facility | Alone | 109 (90) | 132 (86) |
|  | With a family member | 12 (10) | 22 (14) |
| External pick up | Yes | 76 (63) | 102 (66) |
| Home delivery | Yes | 61 (50) | 87 (56) |
| Medication packaging | One bottle for each month | 54 (45) | 63 (41) |
|  | One larger bottle with several months in it | 23 (19) | 27 (18) |
|  | An unmarked (blank) container | 9 (7) | 18 (12) |
|  | A container with instructions on it | 10 (8) | 11 (7) |
|  | A blister pack | 9 (7) | 15 (10) |
|  | Any kind of packaging is fine | 16 (13) | 20 (13) |
| Provider choice | Doctor or clinical officer | 24 (20) | 23 (15) |
|  | Nurse | 91 (75) | 123 (80) |
|  | Counsellor | 4 (3) | 6 (4) |
|  | Community health worker | 2 (2) | 2 (1) |
| More information | More | 58 (48) | 80 (52) |
|  | The same | 59 (49) | 66 (43) |
|  | Less | 4 (3) | 8 (5) |
| More counselling | More | 59 (49) | 82 (53) |
|  | The same | 58 (48) | 67 (44) |
|  | Less | 4 (3) | 5 (3) |
| Information format | Written material (brochure or information sheet) | 55 (45) | 60 (39) |
|  | Class/group session in community (not at clinic) | 5 (4) | 14 (9) |
|  | Class/group session with provider at clinic | 17 (14) | 29 (19) |
|  | One-on-one session with provider at clinic | 71 (59) | 72 (47) |
|  | Social media (e.g. Facebook, Twitter) | 16 (13) | 31 (20) |
|  | Community group in my community | 2 (2) | 6 (4) |
|  | Radio or TV | 27 (22) | 40 (26) |
|  | Videos I can watch online at home | 10 (8) | 13 (8) |
|  | Text messages on my phone | 62 (51) | 77 (50) |
|  | Links to websites that I can browse in my own time | 14 (12) | 35 (23) |
|  | Other specify | 1 (1) | 1 (1) |
| Facility care | As good as | 95 (79) | 106 (69) |
|  | Better than | 22 (18) | 41 (27) |
|  | Worse than | 4 (3) | 7 (5) |
