# Supplementary material for: Characteristics and six-month viral load suppression of clients presenting with advanced HIV disease in South Africa
Source: PLOS Glob Public Health. 2025 Sep 23;5(9):e0004927. doi: 10.1371/journal.pgph.0004927 (PMC12456826; doi:10.1371/journal.pgph.0004927)
Supplement: S4 Table — (DOCX) [file pgph.0004927.s004.docx]

Supplementary table 4. Healthcare resource use among AHD clients stratified by sex

| Variable | Level | Male | Female |
| --- | --- | --- | --- |
| N (%) |  | 121 (44) | 154 (56) |
| Cotrimoxazole therapy | No | 107 (88) | 143 (93) |
|  | Yes | 14 (12) | 11 (7) |
|  |  |  |  |
| TB preventive therapy | No | 26 (21) | 36 (23) |
|  | Yes | 95 (79) | 118 (77) |
| Number of clinic visits in the first 6 months of ART among clients continuously in care | < 6 Visits | 80 (68) | 92 (62) |
|  | ≥6 Visits | 38 (32) | 56 (38) |
| Documented 6 months viral load | No | 42 (35) | 36 (23) |
|  | Yes | 79 (65) | 118 (77) |
