# Supplementary material for: Characteristics and six-month viral load suppression of clients presenting with advanced HIV disease in South Africa
Source: PLOS Glob Public Health. 2025 Sep 23;5(9):e0004927. doi: 10.1371/journal.pgph.0004927 (PMC12456826; doi:10.1371/journal.pgph.0004927)
Supplement: S5 Table — (DOCX) [file pgph.0004927.s005.docx]

Supplementary table 5. Healthcare resource use among AHD clients stratified by ADH disease as defined by CD4 <200 cells/ µL or WHO stage 3 or 4 conditions and CD4 <200 cells/ µL only

| Variable | Level | Symptomatic AHD clients | Asymptomatic AHD clients |
| --- | --- | --- | --- |
| N (%) |  | 41 (15) | 234 (85) |
| Cotrimoxazole therapy | No | 33 (80) | 217 (93) |
|  | Yes | 8 (20) | 17 (7) |
|  |  |  |  |
| TB preventive therapy | No | 22 (54) | 42 (18) |
|  | Yes | 19 (46) | 192 (82) |
| Number of clinic visits in the first 6 months of ART among clients continuously in care | < 6 Visits | 25 (61) | 148 (66) |
|  | ≥6 Visits | 16 (39) | 77 (34) |
| Documented 6 months viral load | No | 13 (32) | 65 (28) |
|  | Yes | 28 (68) | 169 (72) |
