# Supplementary material for: Characteristics and six-month viral load suppression of clients presenting with advanced HIV disease in South Africa
Source: PLOS Glob Public Health. 2025 Sep 23;5(9):e0004927. doi: 10.1371/journal.pgph.0004927 (PMC12456826; doi:10.1371/journal.pgph.0004927)
Supplement: Attachment — Submitted filename: PLOS GPH Reviews_HAF.docx [file pgph.0004927.s007.docx]

**Characteristics and six-month viral load suppression of clients presenting with advanced HIV disease in South Africa**

**Overall Comments**

Well-written article contributing key insights on patient characteristics and six-month viral suppression with a focus on those presenting comparison in clinical outcomes, care engagement, and service preferences between patients with advanced HIV disease and those without. I have a few comments on this article. Please see the sectional comments below

**Title:** The topic is well-suited for the journal.

**Abstract:**

- Methods: Include the study design. Was it an observational cohort, etc.?
- Conclusion: The authors only mention guideline updates to address ADH needs. How about other findings, such as implications of low viremia and service preferences?

**Introduction**

- Lines 70-71-No need for parentheses
- Lines 77-78: Was there a comparison group in the Sub-Saharan African study?
- The last paragraph describes the focus of the paper. It could read better if you also include the unique contribution of your analysis compared to other studies.

**Methods**

- What informed the categorization of the VL suppression categories? Is it based on the guidelines, and why were these thresholds chosen? Include the reason.

**Discussion and conclusion**

- Lines 272-282: The author acknowledges a higher rate of low viremia but does not discuss the clinical and policy implications for managing low viremia
- Lines 291-297: How many patients were likely to report hypertension? Any implications?
- Lines 298-309: What can be done about clients who face barriers to clinic visits?
- Limitations: The authors acknowledge missing CD4 and VL baseline data; however, the root cause is not mentioned.
- Conclusion: Provide details on the targeted interventions and differentiated care models, as these are more general.
