# Supplementary material for: Characteristics and six-month viral load suppression of clients presenting with advanced HIV disease in South Africa
Source: PLOS Glob Public Health. 2025 Sep 23;5(9):e0004927. doi: 10.1371/journal.pgph.0004927 (PMC12456826; doi:10.1371/journal.pgph.0004927)
Supplement: Attachment — Submitted filename: Responses to Reviewer.docx [file pgph.0004927.s009.docx]

**RESPONSES TO REVIEWER COMMENTS for PGPH-D-25-00852: Characteristics and six-month viral load suppression of clients presenting with advanced HIV disease in South Africa**

**REVIEWER #1:**

Many thanks for the opportunity to review this paper. I believe that it describes important research that aims to improve outcomes for those with AHD. My comments to the paper are as follows:

**Introduction**

1. Line 81-83 – perhaps rephrase or clarify why medication adherence and viral suppression is challenging for people with AHD compared to other people with HIV? Apart from potentially having more complex treatment regimens (presumably due to treatment of opportunistic infections), it is not clear why having AHD makes these goals harder?

**Response**: Thank you for raising this; we have added text to explain this further in lines 88 to 97:

*“**ART retention and adherence may be more challenging for people with AHD for three reasons. First, almost by definition, developing AHD suggests that the patient faces barriers to starting or remaining on treatment, relative to those who present for treatment without AHD. These barriers, which may involve transport, employment, fear of stigma, or many other considerations, may not go away after ART is (re-) started. Second, the complexity of treatment regimens and increased pill burden and visit requirements associated with managing the opportunistic infections (OIs) or comorbidities that help define AHD may contribute to treatment fatigue and missed ARV doses. And third, patients with OIs may face challenges in taking or swallowing antiretroviral (ARV) medications due to symptoms such as oral or oesophageal lesions or may experience poor drug absorption caused by gastrointestinal complications (9,10).”*

**Methods**

2. It may be worth mentioned why data from Zambia was not used in this study.

**Response:** Unfortunately, we were not able to access laboratory results of the PREFER cohort enrolled in Zambia and were thus unable to define the AHD cohort using the same approach as we did with the South African participants.

**We have added a sentence in the methods section (line 155 to 157 )**

“*We were unable to access laboratory results for participants enrolled in the PREFER cohort in Zambia, and therefore could not define advanced HIV disease (AHD) using the same criteria applied to South African participants.”*

3. How was re-initiation of ART defined? In Table 1 you refer to self-reported re-engagment in care after a period of disengagement or interruption – was there any time period specified for being out of care (either minimum, maximum or both)?

**Response:** Thank you for raising this; We have added this clarification to the Methods section of the manuscript (see lines 181-187).

*“**We defined re-initiation of ART as self-reported re-engagement in HIV care following an interruption in ART for a period of at least 90 days. This threshold was selected based on existing literature and programmatic definitions of disengagement from HIV care, which commonly use a 90-day threshold to distinguish between temporary non-adherence and more sustained treatment interruption. No maximum time period was specified. Participants who reported being out of care for less than 90 days were not classified as having re-initiated ART, as they were not considered to have disengaged from care per our study definition.”*

Results

4. It would be useful to show the number with measured CD4 and measured WHO stage. Your text mentioned that only 3% met both criteria for AHD (CD4 and WHO stage), but this may be due to low ascertainment of WHO stage? This relates to the authors final point in the conclusion regarding early detection of AHD, and the data may be relevant for regions where access to CD4 testing is especially limited.

**Response:** Thank you for pointing this out. We did in fact have similar ascertainment for CD4 count (n=938) and WHO stage (n=900). We have added text reporting these frequencies for clients with documented baseline WHO staging and CD4 count (line 218 to 220):

*“Of the 1,098 individuals enrolled in the PREFER survey, 938 had a documented baseline CD4count, and 900 had WHO stage recorded at initiation. All clients without baseline CD4 count or WHO staging recorded at baseline were excluded from further analysis.”*

5. Table 2 shows “How often client comes to this clinic for services for additional chronic services”. Please rephrase this to clarify. Is it possible to clarify if these additional chronic services are at separate appointments with other healthcare workers or would the same clinician at the facility see the client for HIV and their comorbidities at the same visit? The question is a little ambiguous as it does not necessarily mean that clients who come for additional chronic services attend the clinic more frequently, so it would be good to clarify this as this is one of your main points in the discussion section.

**Response:** This is a valid point, thank you. In South Africa, integration of services varies by facility, and chronic care and routine HIV visits are not always aligned. We have revised the text accordingly:

*“How often client comes to this clinic for services for additional chronic services other than HIV care”*

The data refer specifically to the number of clinic visits for the management of comorbid chronic conditions (e.g., hypertension, diabetes), and we did not track whether these visits occurred with the same clinician or in the same appointment as HIV care. We acknowledge that this distinction was not explicitly stated. The frequency of visits reported does not necessarily imply that the client received all services (HIV and comorbidities) during the same visit. Some patients may have had separate visits for their HIV care and their other chronic conditions, while others may have received integrated care in a single visit with the same clinician. Unfortunately, we did not collect data specifically on whether these services were provided during separate visits or as part of a combined visit with a single provider.

We have updated Table 2 to reflect this distinction more clearly. We’ve also updated the Discussion (lines 385–390) to address the implications of whether or not services were integrated during the same visit and how this may affect our interpretation of visit frequency and service utilization.

6. Table 2 – make it clear that the proportion of clients that have been treated for TB is calculated using the number ever diagnosed with TB

**Response:** Thank you for pointing this out. We have clarified the question in table 2 to reflect that the proportion of clients treated for TB is calculated using the total number of individuals ever diagnosed with TB, not just those currently receiving treatment

*“Of the clients ever diagnosed with TB, how many were treated .”*

7. Do you have data on which ART regimen participants were receiving, and if not specifically for participants could you describe what proportion of clients at facilities are on DTG-based ART in the methods section, presumably most of them, given the timeframe of the study? This may be relevant as DTG-based ART can lead to more rapid viral suppression so those with low-level viremia could in theory be those on non-INSTI based ART.

**Response:** Yes, we have added this data to Table 1 and also clarified in the results section (line 262 to 263).

**“***Among AHD clients with low-level viremia, 91% were on a Dolutegravir-based regimen, compared to 87% of the non-AHD cohort with low-level viremia.****”***

8. Among those with AHD, were the differences in preferences or resource utilization by sex?

**Response:** Yes, we noted that a higher proportion of females (77%) had a documented 6-month viral load compared to males (65%). The data reveals other notable differences in preferences between males and females. A high proportion of females (44%) prefer visits before 8 am, compared to males (30%). Additionally, males (59%) have a stronger preference for one-on-one sessions with a provider at the clinic than females (47%). When it comes to facility care, a higher percentage of males (79%) rate the care as "as good as expected" compared to females (69%), with more females (27%) rating it as "better than expected". These differences highlight variations in visit timing, preferred care formats, and perceptions of the quality of care between the two genders. We have added Supplementary table 2 and 3, and included the text in results section and discussion sections. *“Symptomatic AHD clients (diagnosed by WHO stage 3/4 and CD4 count <200 cells/µL) and those diagnosed with CD4 count <200 cells/µL alone reported differences in service delivery expectations and preferences. Clients with CD4 <200 cells/µL were more likely to prefer the clinic every month (22% vs. 15%) and prefer receiving medication in 1-month intervals (20% vs. 12%). They also preferred their visits to be early in the month (41% vs. 22%). In contrast, symptomatic clients (WHO stage 3/4 and CD4 <200 cells/µL) were more likely to prefer external pick-up services (67% vs. 51%) (supp. table 5)*.”

see lines  274-280 and discussion 386 to 406.

Discussion

9. Line 261 – The authors state that “the higher frequency of healthcare visits for additional non-HIV services and prior TB diagnoses among AHD clients reflects the severe burden of comorbid conditions in this group…”. As mentioned above, I think this needs further clarification. Your results actually show a similar number of visits for AHD and non-AHD clients (table 5). It is not clear whether AHD clients are attending facilities with the same frequency but use more services during their visits, or whether they visit the clinic more frequently.

**Response**: Thank you for catching this inconsistency. Upon reviewing the data, we would like to clarify the point regarding healthcare visits and the burden of comorbid conditions in AHD clients. In terms of clinic visits, there is only a 4% higher proportion of visits in the AHD group compared to the non-AHD group, with the median number of visits being the same across both groups. This suggests that resource utilization in terms of visits at HIV clinics does not appear to be significantly higher for clients with AHD.

However, it is important to note that the data available does not capture other forms of healthcare utilization that may contribute to the overall burden for AHD clients. These could include access to oncology services, medical outpatient departments (OPD), hospitalizations, and other specialized care that may not be reflected in the dataset. Therefore, while the data does not show an increase in visit frequency at HIV clinics, the additional resource utilization burden may lie in these other areas, which were not captured in our study.

This is a limitation of our study, and we suggest that future research should incorporate data on non-HIV visits and hospitalizations to provide a more comprehensive understanding of the healthcare burden for AHD clients. Such data would allow for a clearer picture of the overall resource utilization, including services outside of routine HIV care.

We rephrased the sentence to reflect a similar number of clinic visits between the 2 groups (Line 319 to 327), and included text in discussion section (line 385 to 390 )

10. The authors also mention higher frequency of prior TB diagnoses. The results (Table 2) do not report prior TB diagnoses but rather ever being diagnosed with TB, which would also include current TB. Since having TB is an indicator for AHD it would be necessary to present results which exclude current TB diagnoses for this statement to be justified.

**Response:** We corrected this to reflect only TB at ART initiation or re-initiation rather than ever been diagnosed with TB (line 242).

11. Line 301-302: Suggest rephrase – The sentence starts off suggestion no difference, but ends stating that the difference was not statistically significant. (line 384)

**Response:** We removed the end part where we mentioned “although this difference was not statistically different”

Lines 298-309: This paragraph is unclear. It starts off suggesting that there was no difference between AHD and non-AHD clients’ visit frequency. There is then a suggestion that those with AHD would have higher visit frequency due to their condition and treatment needs. This statement does not mention the comorbidities and the extra services that AHD clients utilized for these that are described earlier in the discussion section, but only mentions the HIV-related OIs and symptoms of AHD as reasons for more frequent visits. I agree with the statements regarding the barriers to more frequent clinic visits especially for the potentially more vulnerable AHD clients, but your results are unclear whether AHD clients do in fact have more frequent visits. The authors suggest differentiating between clients who are ill and those who are asymptomatic as a way to improve AHD service delivery. To support this statement, would it be possible to disaggregate your results between these 2 groups for service preferences and utilization?

**Response:** We have added a supplementary table with a stratified analysis of symptomatic clients and those with CD4 count <200 cells/µL. *(See line 299 to -302 and 274 to 280) Discussion (line 329-340)*

*“**Symptomatic clients (diagnosed by WHO stage 3/4 and CD4 count <200 cells/µL) and those diagnosed with CD4 count <200 cells/µL alone reported differences in service utilization and service delivery expectations and preferences. Clients with CD4 <200 cells/µL alone were slightly more likely to prefer the clinic every month (22% vs. 15%) and prefer receiving medication in 1-month intervals (20% vs. 12%). They also preferred their visits to be early in the month (41% vs. 22%). In contrast, symptomatic clients (WHO stage 3/4 and CD4 <200 cells/µL) were more likely to prefer external pick-up services (67% vs. 51%). A slightly higher proportion of CD4 <200 cells/µL clients (93% vs. 80%) were not receiving cotrimoxazole therapy than were symptomatic clients.*

**REVIEWER #2:**

Overall Comments

Well-written article contributing key insights on patient characteristics and six-month viral suppression with a focus on those presenting comparison in clinical outcomes, care engagement, and service preferences between patients with advanced HIV disease and those without. I have a few comments on this article. Please see the sectional comments below.

Title: The topic is well-suited for the journal.

Abstract:

1. Methods: Include the study design. Was it an observational cohort, etc.?

**Response:**  This was an observational cohort study. We have noted this in the abstract methods text (line 35 to 36):

*“****This observational cohort collected socio-demographic data, HIV care history, and service delivery preferences, and linked surveys to routine medical records and described client characteristics using descriptive statistics.”***

2. Conclusion: The authors only mention guideline updates to address ADH needs. How about other findings, such as implications of low viremia and service preferences?

**Response**: Thank you for pointing this out. We have expanded the conclusion text to include implications for all findings including persistent low-level viremia (54 to 59):

***“****AHD remains a key barrier to optimal ART outcomes. Clients with AHD experienced poorer viral suppression despite similar retention rates, highlighting the need for early detection, tailored clinical support, and strengthened monitoring. Updating ART guidelines to address AHD-specific needs is critical to improving outcomes in this high-risk population.”*

Introduction

3. Lines 70-71-No need for parentheses

**Response:** Noted – we have removed parentheses (line 74 to 75)

4. Lines 77-78: Was there a comparison group in the Sub-Saharan African study?

**Response:** Regrettably, a non-AHD comparison group was not available for inclusion in our analysis.

5. The last paragraph describes the focus of the paper. It could read better if you also include the unique contribution of your analysis compared to other studies.

**Response:** Thank you for this comment. We have expanded the text in line 117-119 to clarify that this study contributes evidence on early ART outcomes disaggregated by AHD status within routine programmatic settings and highlights the potential impact of baseline sociodemographic and clinical factors as well as client preference on early treatment outcomes.

Methods

6. What informed the categorization of the VL suppression categories? Is it based on the guidelines, and why were these thresholds chosen? Include the reason.

**Response:**  In the South African National Department of Health adherence guidelines, HIV viral load suppression categories for individuals on antiretroviral therapy (ART) are defined as follows: undetectable (viral load below 50 copies/mL), suppressed (detectable but less than or equal to 1,000 copies/mL), and unsuppressed (greater than 1,000 copies/mL). Here, we used these thresholds but refer to the categories as suppressed, low-level viremia and unsuppressed. We have added the following text in the methods section to clarify this:

*“In line with the South African National Department of Health adherence guidelines, HIV viral load suppression categories for individuals on ART are defined as follows: undetectable (viral load <50 copies/mL), suppressed (detectable but ≤1,000 copies/mL), and unsuppressed (>1,000 copies/mL). For the purposes of this analysis, we adopted these thresholds but refer to the categories as suppressed, low-level viremia, and unsuppressed (15).”(line 175 to 179)*

Discussion and conclusion

7. Lines 272-282: The author acknowledges a higher rate of low viremia but does not discuss the clinical and policy implications for managing low viremia

**Response**: Thank you for pointing this out. We have added text describing both the clinical and policy implications for managing low level viremia (line 354 to 352):

***“****Managing low-level viremia (LLV) comes with both clinical and policy implications. On the clinical side, it may indicate that patients need closer monitoring, extra support to stick to their treatment, and/or a change in their medication to avoid drug resistance and treatment failure. From a policy perspective, clear guidelines are needed to decide when to step in, along with enough resources for extra testing and support programs. It is also important to improve data systems like TIER.Net so that cases of low-level viremia are properly tracked and to ensure that healthcare workers are trained to recognize and manage it. Addressing these issues is key to helping patients to achieve favourable treatment outcomes and keeping HIV treatment effective overall.”*

8. Lines 291-297: How many patients were likely to report hypertension? Any implications?

**Response:** Hypertension was reported among 56% of non-AHD clients and 46% of AHD clients; we added the proportions to the text (line 362) and we added text in discussion section (line 368 to 379)

“*Notably, while tuberculosis was more prevalent among AHD clients, hypertension was more frequently reported among clients without AHD. This finding aligns with known immunological and metabolic consequences of HIV infection at different stages of disease progression. Higher rates of hypertension among clients without AHD may be attributable to chronic immune activation and systemic inflammation associated with long-term HIV infection and antiretroviral therapy (ART), even in the absence of advanced disease.*

*These findings underscore the importance of integrated and differentiated care models. While TB screening and treatment remain central to care for clients with AHD, the high burden of hypertension among those without AHD highlights the need for routine screening and management of non-communicable diseases (NCDs) within HIV care programs. Integrated models that address both communicable and non-communicable comorbidities have been shown to improve outcomes and are increasingly relevant as HIV populations age and live longer on ART.”*

9. Lines 298-309: What can be done about clients who face barriers to clinic visits?

**Response**: We have added the following text suggesting service delivery models that can reduce barriers to clinic visits: (Line 395-399):

*“To address these challenges* *tailored service delivery models, such as multi-month dispensing and community ART groups, home delivery of ART, mobile clinics, workplace-based services, and fast-track refills, may reduce barriers to clinic visits and improve access to care, particularly for older, employed men who often present for ART initiation with advanced HIV disease(24,25).”*

10. Limitations: The authors acknowledge missing CD4 and VL baseline data; however, the root cause is not mentioned.

**Response**: We have added the possible root causes of missing baseline CD4 count and VL. (line 438 to 446)

*The missing* ***CD4 count*** *and* ***viral load (VL)*** *baseline data can be attributed to several factors. For* ***CD4 count****, testing is typically done at* ***ART initiation****, but for clients re-engaging in care after a short treatment interruption, a repeat CD4 test may not always be conducted immediately, especially if the client is assessed clinically. For* ***VL testing****, it is scheduled at* ***3 or 6 months*** *after ART initiation, and missing data may occur if clients are* ***not retained in care*** *long enough to reach this time point. Moreover,* ***data linkage issues****, such as duplicate files or mismatches between records, can also lead to missing results. These factors highlight the real-world challenges of HIV care, contributing to gaps in data that were beyond the scope of this study but are important to consider in future research.*

11. Conclusion: Provide details on the targeted interventions and differentiated care models, as these are more general.

**Response**: Thank you for this comment. We have expanded the discussion text describing potential interventions to improve outcomes for clients with advanced HIV: (See line 463-482)

***“****Differentiated care models and complementary interventions can further strengthen early HIV diagnosis, facilitate rapid ART initiation, support the management of comorbidities, and enhance adherence and retention in care. Expanding and targeting HIV testing through community-based and provider-initiated approaches can improve early case detection, particularly among underserved populations (26). Enabling same-day ART initiation by improving facility readiness and using simplified regimens significantly reduces treatment delays and improves early viral suppression and retention outcomes (23,27). Integrating care for opportunistic infections such as tuberculosis and cryptococcal meningitis, as well as non-communicable diseases, allows for more comprehensive care for individuals with complex clinical needs(28). The use of point-of-care diagnostics, such as rapid CD4, CrAg, and TB-LAM tests, facilitates timely identification and management of clients with AHD. Digital adherence tools (e.g., SMS reminders, mobile applications) and peer support models have shown promise in improving adherence and engagement in care (29). Multi-month dispensing, community ART groups, and fast-track refill systems, improve retention and patient satisfaction, especially among working-age men who are disproportionately affected by AHD (30) (31). Although many of these interventions are in place to varying degrees, strengthening the health system through training, supportive supervision, and robust health information systems is essential to ensure consistent implementation and improved clinical outcomes.”*
